# Supplementary material for: A Survey of Wild Indigenous Cryptostylis ovata Orchid Populations in Western Australia Reveals Spillover of Exotic Viruses
Source: Viruses. 2025 Jan 14;17(1):108. doi: 10.3390/v17010108 (PMC11769163; doi:10.3390/v17010108)
Supplement: Supplementary file 1 [file viruses-17-00108-s001.zip › viruses-3392944-supplementary.pdf]

Table S1. Primer pairs used to confirm presence of bean yellow mosaic virus (primer names beginning BYMV) and Ornithogalum mosaic virus (primer names beginning OrMV) by PCR. The primer names also contain information on the name of the genes to which they bind and whether they are forward (F) or reverse (R) primers.

Primer name	Primer sequence 5'>3'	Amplicon size
BYMVP3F	GCAGCACTTGAGGGAGCTAA	518 bp
BYMVCIR	GTTGCGCATTAGCTCTCGTG	
BYMVVPgF	CGCCACACAAACCTCTGTTG	604 bp
BYMVNIAR	CACTGCCCAGCTACTGTTGA	
BYMVNIAF	TCAACAGTAGCTGGGCAGTG	457 bp
BYMVNIBR	GGAAAAATTTTTCTGCTTCATCG	
BYMVNIBF	ATGCAGCAGAGAAGCTTGGT	567 bp
BYMVNIB2R	GGAAACGACACACAAGCACC	
BYMVNIB2F	GGTGCTTGTGTGTCGTTTCC	517 bp
BYMVCPR	TTGGCGGAATGTTGGCTTTG	
OrMVP1F	CTGACATTGCTCAGCCCAGA	450 bp
OrMVHCR	CAAGGTCTGTCACGTCTCCC	
OrMVHCF	TCGCCATCGAACGAGAGAAG	450 bp
OrMVHCR	TACCGATTGCAAGCTCACGT	
OrMVCIF	GGACTGCCGGTTATGACACA	450 bp
OrMVCIR	AGGGATGGCAATTGGATCCG	
OrMVNIBF	GAACTTTCACAGCAGCACCC	450 bp
OrMVNIBR	GGATGTCCAGTTTCGCGAGA	
OrMVCPF	GTCCTCAATCTGCAGCACCT	585 bp
OrMVCPR	CATGTCACGACGAACATCGC	
